# Rapid differentiation of PEDV wild-type strains and classical attenuated vaccine strains by fluorescent probe-based reverse transcription recombinase polymerase amplification assay

**DOI:** 10.1186/s12917-020-02424-1

**Published:** 2020-06-22

**Authors:** Zhilin Wang, Xuerui Li, Youjun Shang, Jinyan Wu, Zhen Dong, Xiaoan Cao, Yongsheng Liu, Xi Lan

**Affiliations:** 1 State Key Laboratory of Veterinary Etiological Biology, Lanzhou Veterinary Research Institute, Chinese Academy of Agricultural Sciences, Lanzhou, 730046 Gansu PR China; 2 College of Veterinary Medicine, Guangdong Provincial Key Laboratory of Prevention and Control for Severe Clinical Animal Diseases, South China Agricultural University, Guangzhou, 510642 Guangdong PR China

**Keywords:** Porcine epidemic diarrhea virus, Recombinase polymerase amplification, Differentiate, ORF1

## Abstract

**Background:**

Porcine epidemic diarrhea virus (PEDV), an intestinal coronavirus that causes acute diarrhea and high mortality in suckling piglets, can result in high economic losses in the swine industry. In recent years, despite the use of China’s current vaccine immunization strategy, multiple types of PEDV strains were still found in immunized swine herds. Our research aims to explore a new rapid differentiation method to distinguish the different types of PEDV strains and assess the safety evaluation of classical attenuated vaccine strains in swine herds.

**Results:**

In the study, a differential one-step quantitative real-time fluorescent reverse transcription recombinase polymerase amplification (real-time RT-RPA) method based on the PEDV universal real-time RT-RPA assay was established according to the ORF1 deletion sequences of three classical attenuated vaccine strains (PEDV attenuated vaccine KC189944, attenuated CV777 and DR13) and five Vero cell-adapted isolates (JS2008, SDM, SQ2014, SC1402, HLJBY), which could effectively differentiate PEDV classical attenuated vaccine strains from wild-type strains (PEDV classical wild strains and variant strains). The detection limits of PEDV RNA in the both PEDV real-time RT-RPA assays were 300 copies within 20 min at 39 °C, and the detection limits of classical attenuated vaccine strain CV777, Vero-cell-adapted isolate JS2008, and PEDV wild-type strain DX were 10^0.5^ TCID_50_/100 μL, 10^1.1^ TCID_50_/100 μL, and 10^1.2^ TCID_50_/100 μL, respectively. Both assays were highly specific for PEDV, showing no cross-reactivity with other enteral viruses.

**Conclusion:**

This RPA method we developed is simple, time-effective, and safe and provides a reliable technical tool for the differential diagnosis and clinical epidemic surveillance of PEDV classical attenuated vaccine strains and wild-type strains.

## Background

Porcine epidemic diarrhea virus (PEDV) is a serious pathogen which is characterized by severe diarrhea, vomiting, and dehydration in pigs [1, 2]. PEDV is an enveloped, single-stranded, positive-sense RNA virus belonging to the alpha coronavirus genus of the Coronaviridae family [1]. The entire genome sequence of PEDV is approximately 28 kb, which consists of seven open reading frames, encoding four structural proteins [spike protein (S, 150–220 kDa), membrane protein (M, 20–30 kDa), envelope protein (E, 7 kDa), and nucleocapsid protein (N, 58 kDa)], and three non-structural proteins (replicases 1a and 1b and ORF3) [3–5]. PED was first reported in the United Kingdom in 1971 [6]. In 1978, PEDV was isolated in Belgium and named CV777 [1]. In China, a diarrheal disease caused by PEDV was first observed in 1973 [7]. Since the winter of 2010, large-scale outbreaks of PED caused by highly pathogenic PEDV strains have resulted in the death of a large number of pigs in south China [8, 9]. Subsequently, this highly pathogenic PEDV strains were discovered in other countries including the United States and South Korea, etc. [10–13]. Since the 1990s, Both inactivated and live attenuated PEDV vaccines have been widely used to prevent dissemination of the virus in Asia, including CV777 strain-based inactivated or attenuated live vaccines and bivalent inactivated vaccine using attenuated PEDV and TGEV in China [14, 15], the KPED-9 and DR13 strain-based attenuated live vaccines in South Korea [11, 16], and the P-5 V strain-based live attenuated vaccine in Japan [17]. However, despite the emergence of China’s current vaccine immunization strategy, multiple types of PEDV strains were still found in immunized swine herds [15, 18–21]. An analysis of PEDV whole-genome differences whose sequences were available in GenBank indicated that four hypervariable regions were present in PEDV, comprising the C terminus of the nsp2 gene and the N terminus of the nsp3 gene, the Spike gene, the open reading frame 3 (ORF3), and the N gene region [22]. These gene mutations might alter the antigenicity of vaccines derived from classical attenuated vaccine strains and consequently resulted in inefficient vaccination in many pig farms [23]. At present, a variety of strains already exists in China, including classical strains (classical wild strains and classical attenuated vaccine strains, genotype 1) [23, 24], highly virulent strains (genotype 2) [24], the S-INDEL-PEDV strains (genotype 1b) [7, 25, 26], recombinant variants of attenuated vaccine strains and wild-type strains [27, 28], and the variant with a large deletion in the S1 N-terminal domain,etc. [29]. Moreover, multiple types of PEDV strains co-existed in the same environment and even co-infected the same pig [23], with a potential risk of recombination between wild-type strain and the classical attenuated vaccine strain [27, 28]. This could potentially result in the enhancement of the recombinant attenuated vaccine strains in virulence and increase the difficulty of identifying different types of strains. Therefore, it was particularly important to establish a new detection method to distinguish PEDV classical attenuated vaccine strains and wild-type strains and to monitor the prevalence of classical attenuated vaccine strains in pigs and evaluate attenuated vaccine safety.

In recent years, the sequence characteristics of the spike (S) gene in PEDV strains, including insertion and deletion in the S gene (S-INDEL) and only the S1 deletion gene, have been used as genetic markers to distinguish to distinguish different types of PEDV strains [25, 29]. The research showed that nucleotide deletions of the ORF3 gene in the Vero cell-adapted attenuated vaccine strain were used to distinguish PEDV attenuated vaccine strains and wild-type strains [16, 30]. Based on the variation characteristics of the S gene and ORF3 gene, many methods including traditional PCR [23], real time RT-PCR [24, 31, 32] and nanoparticle-assisted RT-PCR [21] have been established. However, there are difficulties in the monitoring, diagnosis and prevention of different types of PEDV strains.

**Fig. 1:**
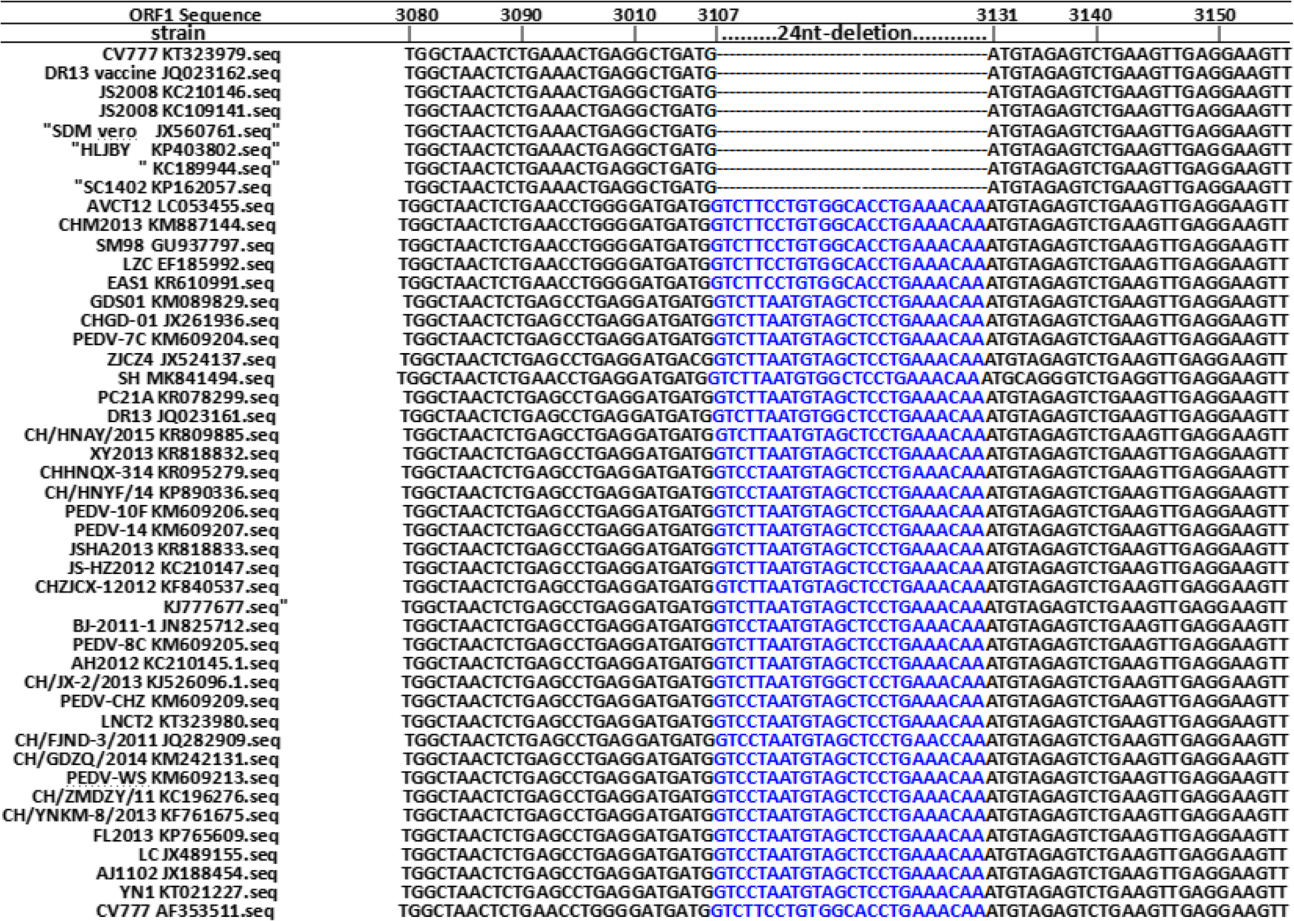
ORF1 regions alignment results of 38 PEDV wild-types strains and 8 Vero cell-adapted strains whose sequences were available in GenBank, and 24-nucleotide deletions in the ORF1 regions of three PEDV classical attenuated vaccine strains and five Vero-cell-adapted isolates

In the present study, compared with the genome sequence of 38 PEDV wild-type strains and 8 Vero cell-adapted strains whose sequences were available in GenBank (Fig. 1 and Table S1), we found that three Vero cell-adapted classical attenuated vaccine strains (PEDV attenuated vaccine KC189944, attenuated CV777 and DR13) derived from classical strains and five Vero cell-adapted isolates (JS2008, SDM, SQ2014, SC1402, HLJBY) have a 24-nucleotide deletion sequence of non-structural protein 3(nsp3) gene in the ORF1. These eight Vero cell-adapted strains were artificially cell-passaged and did not naturally exist in the field unless they were used as attenuated live vaccines to be inoculated into pigs. Based on these discoveries, a real time RT-RPA method was developed to effectively differentiate PEDV classical attenuated vaccine strains from wild-type strains. This method contained not only a PEDV universal real-time RT-RPA assay targeting the nucleocapsid gene that can identify all types of PEDV strains but also included another PEDV vaccine real-time RT-RPA assay targeting the 24-nucleotides deletion sequence in the ORF1 of three classical attenuated vaccine strains that specifically identified PEDV classical attenuated vaccine strains. This method was shown to be an excellent alternative tools for the preliminary differentiation of PEDV classical attenuated vaccine strains and wild-type strains, with potential use as a diagnostic method in clinical samples.

## Results

### The specificity, sensitivity, and repeatability analysis of the PEDV real-time RT-RPA method

**Fig. 2:**
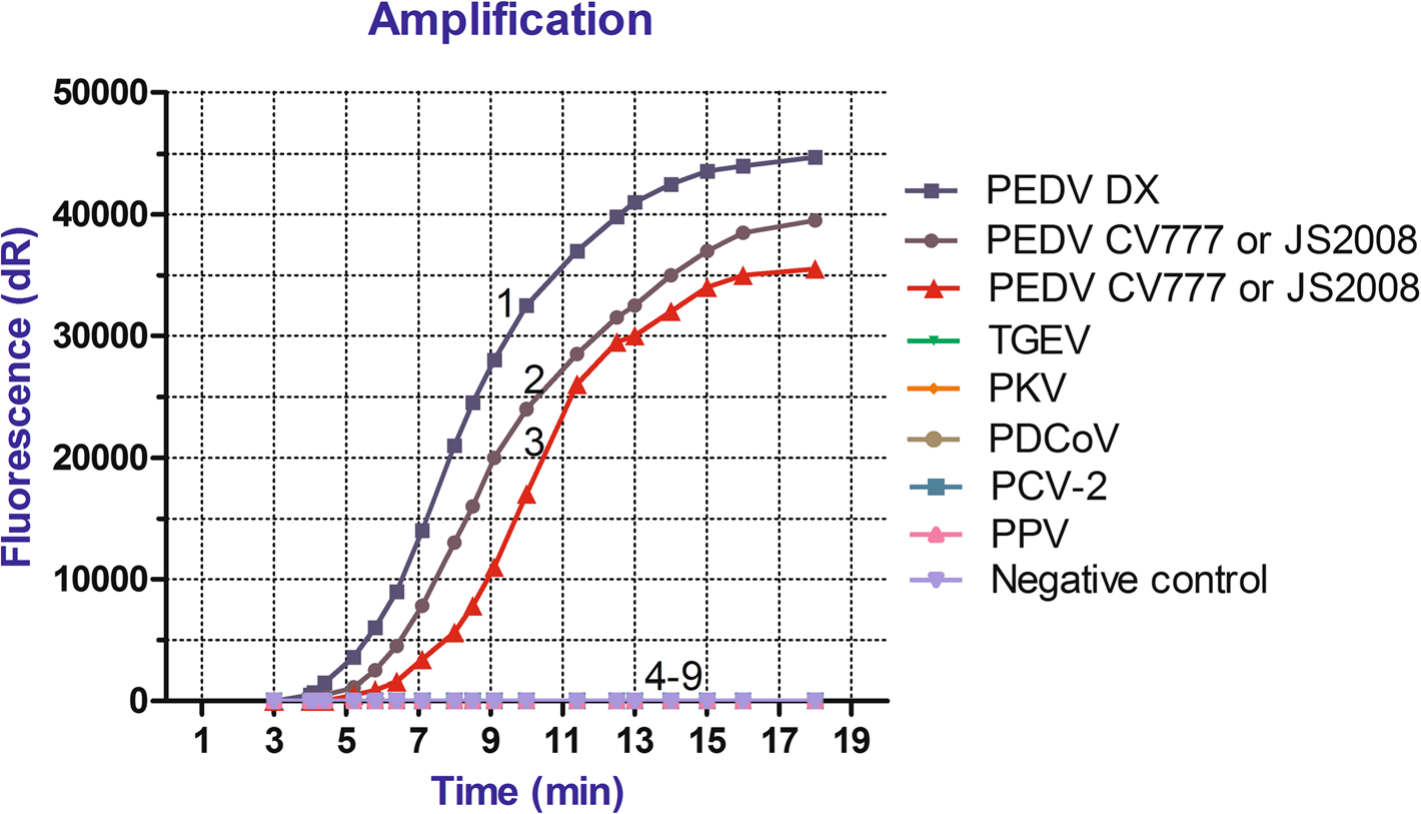
The specificity of both PEDV universal real-time RT-RPA and PEDV vaccine real-time RT-RPA: 1, PEDV DX RNA was amplified in the PEDV universal real-time RT-RPA; 2, PEDV CV777 RNA and JS2008 RNA were amplified in the PEDV universal real-time RT-RPA; 3, only PEDV CV777 RNA and JS2008 RNA was amplified in the PEDV vaccine real-time RT-RPA; 4–9: TGEV, PKV, PDCoV, PCV-2, PPV, and negative control were not amplified in the both PEDV real-time RT-RPAs

As shown in Fig. 2, PEDV wild-type strain DX, classical attenuated vaccine strain CV777, and Vero-cell-adapted isolate JS2008 showed fluorescent signals in the PEDV universal real-time RT-RPA assay, and only the PEDV classical attenuated vaccine strain CV777 and Vero-cell-adapted isolate JS2008 showed fluorescent signals in the PEDV vaccine real-time RT-RPA assay. No fluorescent signals were obtained for TGEV, PCV-2, PDCoV, PPV or PKV, indicating the high specificity of the two assays.

**Fig. 3:**
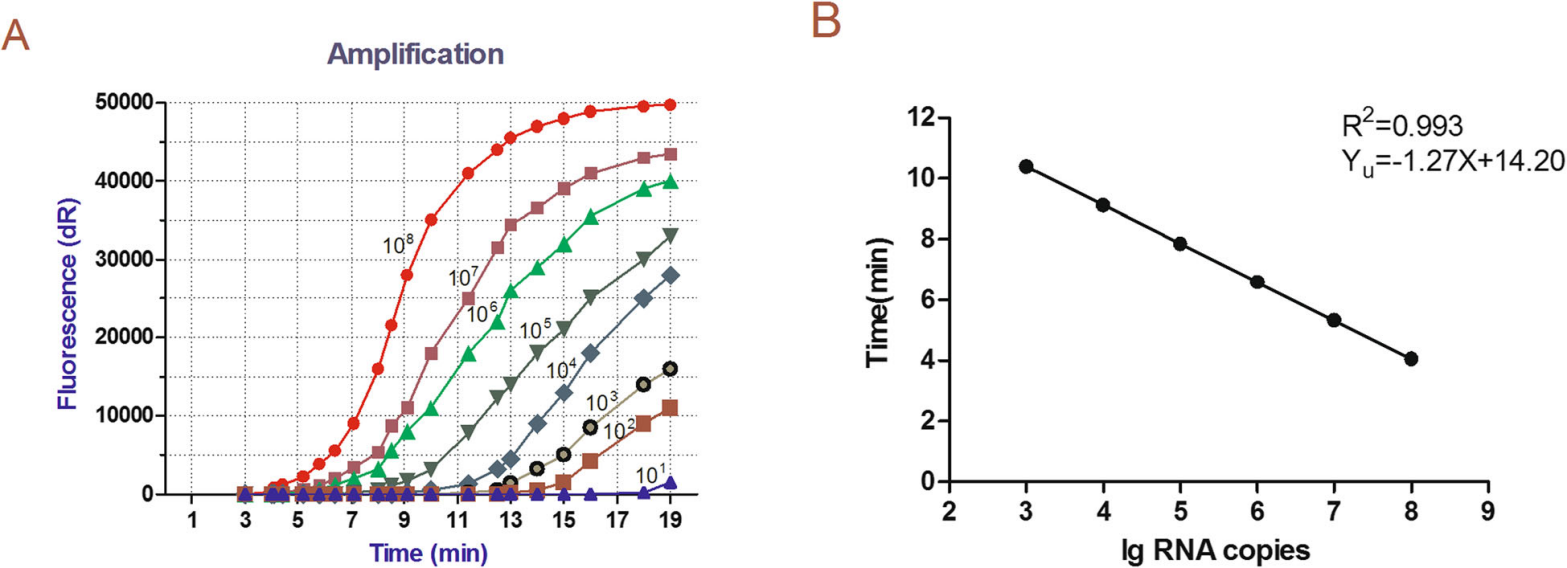
**a** Sensitivity of PEDV universal real-time RT-RPA. Fluorescence development over time using a dilution range of 10^8^–10^1^ copies of the RNA standard. **b** Standard curves of PEDV real-time RT-RPA. Linear regression of the data providing a formula of Yu = −1.27x + 14.20 (R^2^ = 0.993) between the corresponding amount of RNA copy numbers and time, when performed by PEDV universal real-time RT-RPA

**Fig. 4:**
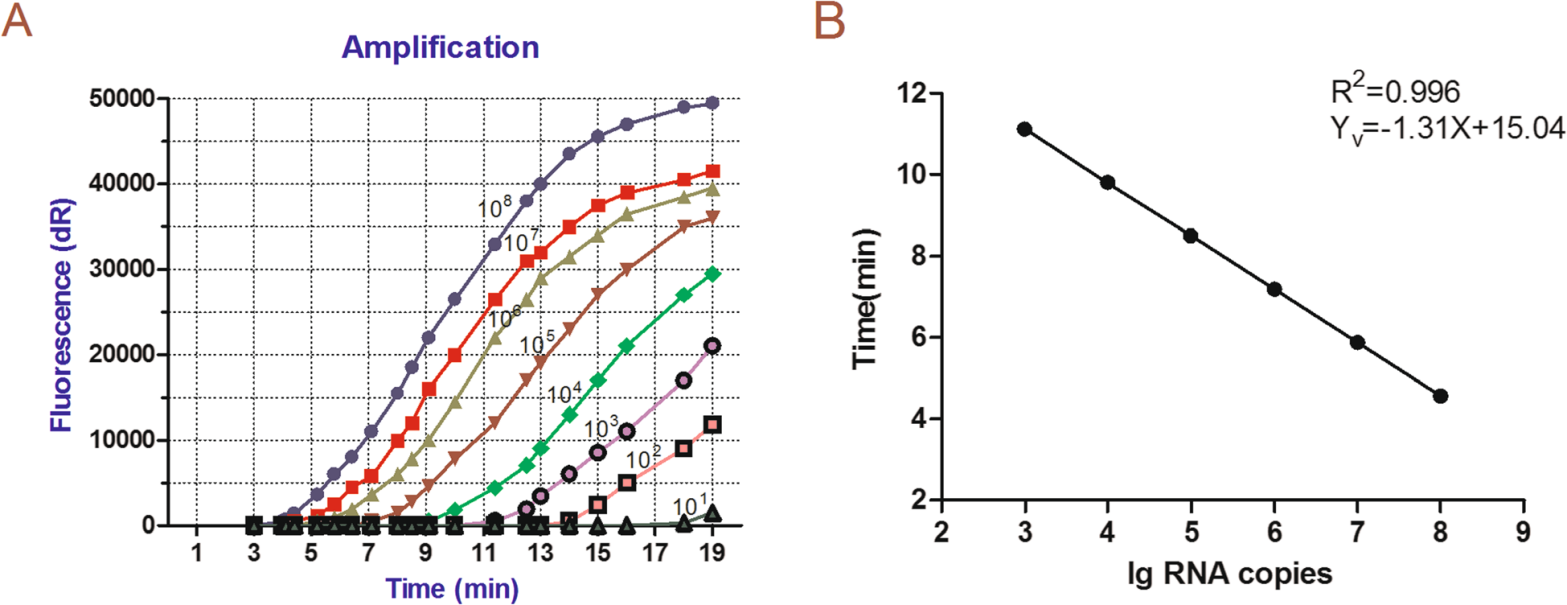
**a** Sensitivity of PEDV vaccine real-time RT-RPA. Fluorescence development over time using a dilution range of 10^8^–10^1^ copies of the RNA standard. **b** Standard curves of PEDV vaccine real-time RT-RPA. Linear regression of the data providing a formula of Yv = − 1.31x + 15.04 (R^2^ = 0.996) between the corresponding amount of RNA copy numbers and time, when performed by PEDV vaccine real-time RT-RPA

PEDV universal real-time RT-RPA and PEDV vaccine real-time RT-RPA standard curves were established using different copy numbers of standard RNA as templates for sensitivity analysis. The detection limits of both the PEDV universal real-time RT-RPA assay (Fig. 3) and the PEDV vaccine real-time RT-RPA assay (Fig. 4) were 3.0 × 10^2^copies/reaction. As shown in Table 1, the detection limits of both PEDV standard RNAs were 3.0 × 10^2^ RNA copies/reaction. Repeatability was evaluated using two standard RNAs of the PEDV attenuated vaccine strain CV777 N gene and the ORF1 region, respectively, with coefficients of variation 0.68–1.47 (Table 1). For the viruses that infect in Vero-E6, the detection limits of PEDV wild-type strain DX, classical attenuated vaccine strain CV777, and Vero-cell-adapted isolate JS2008 were 10^1.2^ TCID_50_/100 μL, 10^0.5^ TCID_50_/100 μL, and 10^1.1^ TCID_50_/100 μL, respectively.

**Table 1:** The sensitivity and repeatability of established PEDV real-time RT-RPA method

Standard RNA	Copy number	Time (mean ± S.D.)^c^	CV%	Standard RNA	Copy number	Time (mean ± S.D.)^c^	CV%
N gene of PEDV CV777^a^	3.0 × 10^8^	4.04 ± 0.041	1.01	ORF1 of PEDV CV777^b^	3.0 × 10^8^	4.56 ± 0.067	1.47
	3.0 × 10^7^	5.31 ± 0.061	1.15		3.0 × 10^7^	5.87 ± 0.071	1.20
	3.0 × 10^6^	6.58 ± 0.075	1.14		3.0 × 10^6^	7.18 ± 0.087	1.21
	3.0 × 10^5^	7.85 ± 0.081	1.03		3.0 × 10^5^	8.49 ± 0.802	0.94
	3.0 × 10^4^	9.12 ± 0.076	0.83		3.0 × 10^4^	9.80 ± 0.068	0.69
	3.0 × 10^3^	10.39 ± 0.108	1.04		3.0 × 10^3^	11.11 ± 0.075	0.68
	3.0 × 10^2^	11.66 ± 0.096	0.82		3.0 × 10^2^	12.42 ± 0.093	0.75
	3.0 × 10^1^	None	None		3.0 × 10^1^	None	None
	3.0 × 10^0^	None	None		3.0 × 10^0^	None	None
	NCT	None	None		NCT	None	None

a. Related to the N gene of PEDV classical attenuated vaccine strain CV777, the Standard RNA as template was used in the PEDV universal real-time RT-RPA assay

b. Related to the ORF1 region of PEDV classical attenuated vaccine strain CV777, the Standard RNA as template was used in the PEDV vaccine real-time RT-RPA assay

c. The reaction procedure was performed by a total of 60 cycles in the real time RT-RPA method (20 s per cycle)

The viral load originally contained in the sample was calculated as Y_u_ = − 1.27x + 14.20 (R^2^ = 0.993)(Fig. 3b) for the PEDV universal real-time RT-RPA assay and Y_v_ = − 1.31x + 15.04 (R^2^ = 0.996) (Fig. 4b) for the PEDV vaccine real-time RT-RPA assay.

### Evaluation of PEDV real-time RT-RPA method, real-time RT-PCR, and one-step RT-PCR assays with clinical samples

**Fig. 5:**
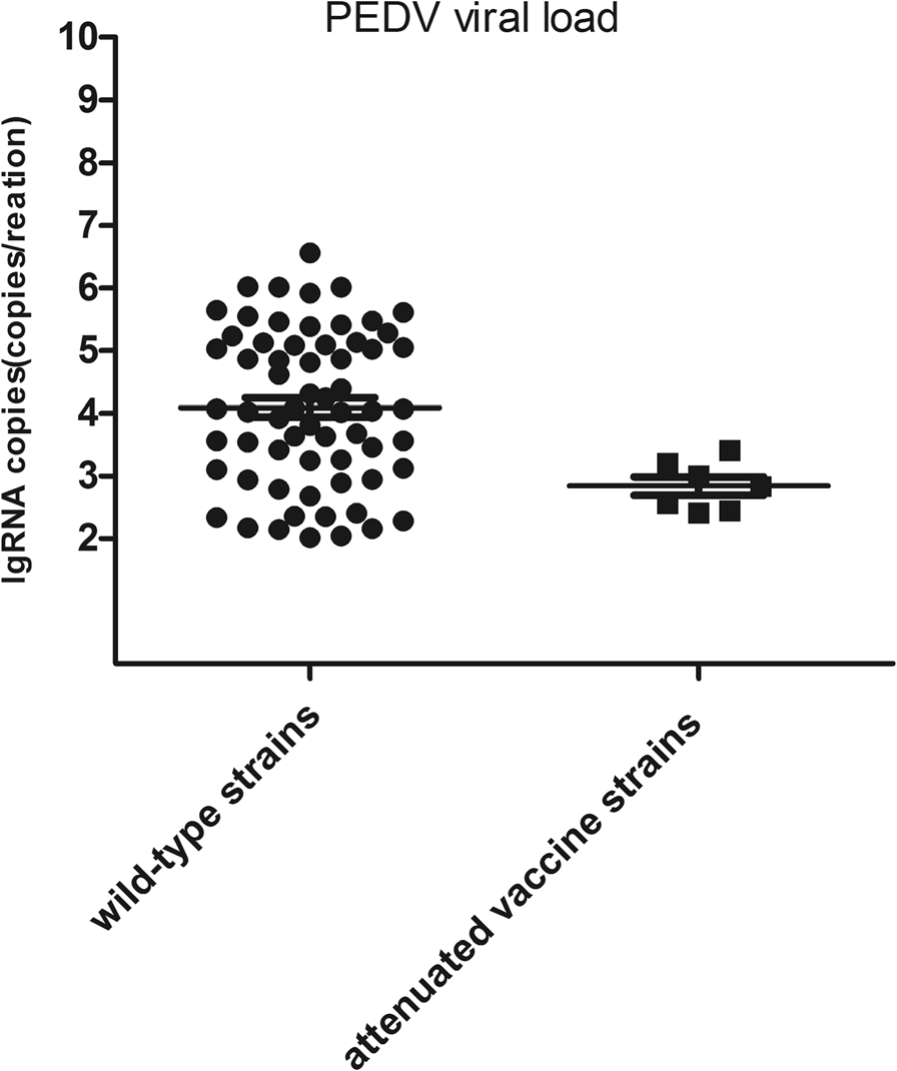
PEDV viral load detection of each PEDV-positive clinical sample by the real time RT-RPA method

**Fig. 6:**
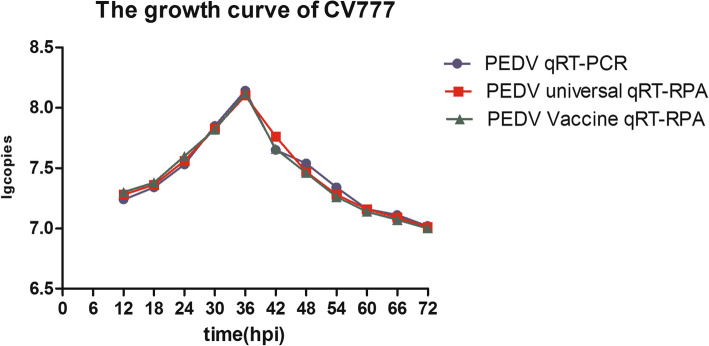
Growth curve of vaccine strain CV777 proliferating within VERO-E6 cells at a multiplicity of infection (MOI) of 0.01 as assessed by PEDV universal real time RT-RPA, PEDV vaccine real time RT-RPA, and PEDV real time RT-PCR assays

To verify the reliability of the established real time RT-RPA method, a total of 80 suspected PEDV samples were assessed by PEDV real-time RT-RPA method, real-time RT-PCR assay, and RT-PCR assay, giving positive rates of 80.00, 81.25, and 77.50% for PEDV wild-type strains, and 8.75, 8.75, and 7.50% for PEDV classical attenuated vaccine strains, respectively (Table 2). Of the 12 samples that tested negative in the RT-PCR assay, 9 samples were negative and the other 3 were positive (one classical attenuated vaccine strain and two wild-type strains) as tested by the real time RT-RPA method, and 8 samples were negative and the other 4 were positive (one classical attenuated vaccine strain and three wild-type strains) as tested by the real time RT-PCR assay. All positive amplified products were sequenced, which confirmed the presence of PEDV in the samples and the viral load of all PEDV positive samples was measured by real-time RT-RPA method in this study (Fig. 5). The PEDV real-time RT-RPA method has highly positive diagnosis agreement with real-time RT-PCR (98.6%) and RT-PCR assays (95.8%). These indicated the high specificity and sensitivity of these assays. In addition, transmissible gastroenteritis virus (TGEV) and porcine kobuvirus (PKV) were detected in the 8 PEDV-negative samples (data not shown). The growth curve for the PEDV classical attenuated vaccine strain CV777 in Vero-E6 cells determined by PEDV real-time RT-PCR, PEDV universal real-time RT-RPA, and PEDV vaccine real-time RT-RPA indicated that viruses replicated rapidly during the first 24 h post-infection (hpi), achieving the highest titer at approximately 36 hpi (Fig. 6). The virus titer gradually decreased because Vero-E6 cells breakdown after 36 h. These results showed that real-time RT-PCR and real-time RT-RPA are two alternative assays for the differentiation and characterization of PEDV properties in Vero-E6 cells.

**Table 2:** Positive samples in PEDV real-time RT-RPA method, real-time RT-PCR assay, and RT-PCR assay

Location in Gansu Province	Samples	qRT-PRA(a)		qRT-PCR(b)		RT-PCR(c)	
		Wild PEDV	Vaccine PEDV	Wild PEDV	Vaccine PEDV	wild PEDV	vaccine PEDV
DingXI	25	23	0	24	0	23	0
JiayuGuan	19	15	2	15	2	14	2
LinXia	23	17	3	17	3	16	2
TianShui	13	9	2	9	2	9	2
total	80	64	7	65	7	62	6
positive rate		80.00%%	8.75%	81.25%	8.75%	77.50%	7.50%

a. Method established in this study

b. Method from Su, et al. 2018 [31]

c. Method from Zhao, et al. 2014 [32]

## Discussion

Phylogenetic tree analysis of PEDV strains in China demonstrated that the entire PEDV genomes evolved into two separate genogroups, GI (classical strains, GI-a and GI-b) and GII (variant strains) [25, 33]. Classical CV777 (accession number: AF353511) and DR13 (accession number: JQ023161) belonged to the GI-a subgroup. The GI-b subgroup included Vero cell-adapted vaccine strains (PEDV attenuated vaccine KC189944, attenuated CV777 and DR13) derived from classical strains, a recombinant Vero cell-adapted isolate (JS2008) of PEDV attenuated vaccine and mutants [27], and four other Vero cell-adapted isolates (SDM, SQ2014, SC1402, HLJBY) [20, 25, 33–36]. PEDV strains in the GI-b subgroup not only have nucleotide variations in the ORF3 or Spike gene [25, 29, 33] but also have 24-nt deletions of the nsp3 gene in the ORF1 region in our study (Fig. 1). These nucleotides deletion in the ORF1 may have occurred in cell-adapted viruses during adaptation and attenuation through serial passage in Vero cells, which is identical to nucleotide deletion of Spike or ORF3 genes in the cell-adapted isolates and attenuated live vaccines [13, 16], and thus would be unlikely to be detected in wild-type strains. Based on the above findings, a differential PEDV real-time RT-RPA method was established to distinguish PEDV classical attenuated vaccine strains from the wild-type strains. This method contained two real-time RT-RPAs, a universal real time RT-RPA assay targeting nucleocapsid gene was used to detect all types of PEDV strains and another PEDV vaccine real time RT-RPA could identity PEDV classical attenuated vaccine strains according to ORF1 24-nucleotides deletion region of three classical attenuated vaccine strains (PEDV attenuated vaccine KC189944, attenuated CV777 and DR13) and five Vero cell-adapted isolates (JS2008, SDM, SQ2014, SC1402, HLJBY) genome sequences, which were compared to 38 PEDV wild-type strains published by GenBank (Fig. 1). The two real-time RT-RPA assays have good specificity, with fluorescent signals only visible for PEDV positive RNA amplicons. The PEDV detection limits of both real-time RT-RPA assays were 10^2^copies, reflecting good sensitivity. Clinical samples tests indicated that the PEDV real-time RT-RPA method has a highly overall agreement with real-time RT-PCR (98.6%) and RT-PCR assays (95.8%) (Table 2), respectively, but require less than half the time of them, suggesting that the real-time RT-RPA method could be used as an alternative detection method. Sequencing results of 7 samples that were positive in the PEDV vaccine real time RT-RPA assay were the same as the nucleotide deletion positions of the ORF1 and ORF3 fragments in classical attenuated vaccine CV777. It is worth noting that among the seven classical attenuated vaccine strains in all samples, six were from the piglets that were orally inoculated with the classical attenuated vaccine CV777, and none of the live PEDV strain was successfully isolated in Vero-E6 cells. This may be because the stool samples contained less viral load or only contains nucleic acids. Due to ORF1 of some vaccine candidates have not been reported, especially the vaccine candidates from highly virulent strains (genotype 2a) emerged after 2010, we are not sure whether these vaccine candidates derived from non-classical attenuated vaccine strains have the same 24 nucleotides deletion of nsp3 gene in the ORF1. If these vaccine candidates do not have the 24 nt deletions pattern, they can not be detected by our method. Similarly, if the wild-type virus may repair the nsp3 gene 24-nt-deletion region of classical attenuated vaccine strain in a co-infection event, the 24 nucleotides-repaired strain not be detected by our method. Nevertheless, commercial vaccines widely used in pig farms of China are developed based on classical attenuated strains, while vaccines derived from non-classical attenuated vaccine candidates are being developed but not yet commercialized, our method is safe, accurate and reliable for the detection and identification of classical attenuated vaccine strains in pig farms. Therefore, our method can be used to distinguish classical attenuated vaccine strains and wild-type strains, while vaccine candidates derived from other non-classical attenuated vaccine strains may not be detected.

This real-time RT-RPA method was established to distinguish between classical attenuated vaccine strains that were artificially vaccinated and wild-type strain during epidemiological surveillance, thereby obtaining more accurate epidemiological data. Additionally, because the attenuated vaccine strain and the wild-type isolates coexist in the same environment, there is a potential risk of genetic recombination between the two strains as well as the enhancement of attenuated vaccine strain virulence. Thus, monitoring the transmission of attenuated vaccine strains and the resultant decline in virus titer could provide technical support for the safety evaluation of attenuated vaccine strains.

Real-time RT-RPA is more rapid than real-time RT-PCR and isothermal loop-mediated isothermal amplification (LAMP) technology. It is also more efficient, requiring only a pair of primers and a low running temperature (30–45 °C) for a short period (20–40 min) [37–39]. This compares with LAMP requirements of 4–6 primers and a high running temperature (60 °C) for 1 h [40–42], and real-time RT-PCR of a pair of primers and a high running temperature (95 °C) for over 1 h [43]. Additionally, there are no melting temperature requirements for RPA primers and probes because their annealing and elongation are enzyme-mediated rather than temperature-driven [39, 44]. Moreover, the combination of RPA and real-time fluorescence quantification with gel electrophoresis results in high simplicity, specificity, and accuracy [38, 39, 45]. Finally, the portability of the assays means that they can be used in the field and in areas where resources are limited [46].

## Conclusion

In Conclusion, we developed a simple, rapid, and reliable real time RT-RPA method for the differentiation of PEDV classical attenuated vaccine strains and wild-type strains. These assays were analyzed using fluorescent dyes and shown to be highly specific and sensitive. They provide a reliable technical tool for the differentiation of the PEDV classical attenuated vaccine strains and wild-type strains, as well as the surveillance of the clinical epidemic status of the disease.

## Methods

### Virus strains and clinical samples

PEDV attenuated vaccine strain CV777 and Vero-cell-adapted isolates JS2008 were passaged in Vero E6 cells. PEDV wild strain DX, transmissible gastroenteritis virus (TGEV), porcine circovirus type 2 (PCV-2), porcine deltacoronavirus (PDCoV), porcine kobuvirus (PKV), and porcine parvovirus (PPV) were maintained in our laboratory. Fecal samples were collected from 80 piglets suspected of being infected with PEDV from five pig farms in Dingxi, Jiayuguan, Linxia, and Tianshui, Gansu Province, China. Clinical samples were centrifuged at 4000 *g* for 15 min, and the supernatant was stored at − 80 °C.

### DNA/RNA extraction

Viral RNA and DNA were extracted using the TaKaRa MiniBEST Viral RNA/DNA Extraction Kit Ver. 5.0 (Takara Co., Ltd., Dalian, China) according to the manufacturer’s instructions, and then quantified using an ND-2000c spectrophotometer (Thermo Scientific, Wilmington, DE, USA). For clinical samples, extracted viral RNA was eluted in 30 μL of RNase-free water. All RNA and DNA templates were stored at − 80 °C until required.

### Primer and probe design

Real-time RPA primers and probes (synthesized by Sangon Biotech, Shanghai, China) were designed and verified by BLAST analysis (https://blast.ncbi.nlm.nih.gov/Blast.cgi) according to the nucleocapsid gene conserved sequence and replicase gene ORF1 region (containing a 24-nucleotide deletion) of the PEDV classical attenuated vaccine CV777 strain (Table 3), and TwistDx (TwistDx, Cambridge, United Kingdom) RPA kit guidelines.

**Table 3:** Sequence of primers and probes for PEDV real-time RT-RPA method

Name	Sequence(5′-3′)	Location	Product size (bp)
F27313	TCGTGAGCTAGCGGACTCTTACGAGATTAC	N,27313-27342^a^	
R27453	GCTGCAGCGTGGTTTCACGCTTGTTCTTCT	N,27453-27482^a^	170 bp
P27375	ATCCAAATGTTGAGCTTCTTGTTTCACAGG (FAM-dT) G (THF) A (BHQ1-dT)GCATTTAAAACTGGG-C3 space	N,27375-27424^a^	
F3330	GTGATGAAGTAGACTCCTCTGACCCTGATA	ORF1,3330-3359^a^	
R3467	CTTAGTAACTGTGGAAGGTGTATCTTTAAT	ORF1,3467-3496^a^	167 bp
P3364	GGCAGATGTGGCTAACTCTGAAACTGAGGC (FAM-dT) GA (THF) GA (BHQ1-dT)GTAGAGTCTGAAGTT-C3 space	ORF1,3364-3413^a^	
F_ORF1_	CACCGATCCTAATCTGCCCG	ORF1,3217-3236^a^	
R_0RF1_	TGGACCAACTCTACCAGCAC	ORF1,3612-3632^a^	415 bp

a. PEDV calssical attenuated vaccine CV777 strain, GenBank accession number KT323979

### Generation of standard RNA

PEDV nucleocapsid gene segments (301 bp, ranging from 27,233–27,533 bp of CV777, GenBank accession number KT323979.1), named PEDV-N/qRT-RPA, were synthesized and inserted into the pGEM-T Easy vector by Genecreate (Nanjing, China) to create a plasmid DNA standard for use in the PEDV universal real-time RT-RPA assay. Another PCR fragment of the PEDV ORF1 region was amplified using primers F_ORF1_/R_ORF1_ (Table 3) from PEDV classical attenuated vaccine CV777 cDNA and named PEDV-V/qRT-RPA. Synthesis of the first-strand PEDV classical attenuated vaccine strain CV777 cDNA was performed by reverse transcription using the PrimeScript™ 1st Strand cDNA Synthesis Kit (Takara, Dalian, China), and the PCR conditions were as follows:95 °C for 5 s, followed by 35 cycles of 95 °C for 5 s, 55 °C for 30 s, and 72 °C for 30 s, with a final extension at 72 °C for 10 min. The amplified PCR product was then linked to the pGEM-T Easy vector to construct the standard plasmid for use in the PEDV vaccine real-time RT-RPA assay. Both plasmids were sequenced by TsingKe Biological Technology, linearized by digestion with *Nde* I (Takara Co., Ltd.), purified using the TaKaRa MiniBEST DNA Fragment Purification Kit Ver. 4.0 (Takara Co., Ltd.), and transcribed in vitro with the RiboMAX Large Scale RNA Production System-T7 (Promega, Madison, WI, USA). Agarose gel electrophoresis was used to verify the length and integrity of transcribed standard PEDV RNA in vitro. The number of standard RNA copies was calculated using an ND-2000c spectrophotometer and the RNA copy number was calculated as follows: (6.02 $\ddot{\mathrm{y}}$ 10^23^ copy number/mole number) × (RNA concentration)/(340 × base number).

### Real-time RT-RPA assay

The real-time RT-RPA assay was performed using TwistAmp® exo RT (TwistDx) in the following assay reaction system: 420 nM (2.1 μL) of each RPA primer (10 μM), 120 nM (0.6 μL) exo probe (10 μM), 14 mM (29.5 μL) rehydration buffer, 1 μL of viral RNA or 4 μL sample RNA, 1 μL of RNase inhibitor, and ddH_2_O to a total volume of 47.5 μL. After mixing, this was added to the recombinase reaction pellet with 2.5 μL 280 mM magnesium acetate and mixed well. The sample was vortexed, briefly centrifuged, and the tubes were immediately placed in the Agilent Technologies Mx3000P thermocycler device (Life Technologies, Carlsbad, CA, USA) to start the reaction at 39 °C for 20 min (20 s per cycle, a total of 60 cycles).

### Real-time RT-PCR assay

Real-time RT-PCR was performed to identify PEDV wild-type strains and classical attenuated vaccine strains in an Agilent Mx3000P thermocycler machine (Life Technologies) using the primers and Probe based on the PEDV Spike gene [31] with the One Step PrimeScript® RT-PCR Kit (Perfect Real Time; Takara Co., Ltd). The assay was performed as follows: 42 °C for 5 min, then 95 °C for 10 s, followed by 40 cycles of 95 °C for 5 s and 60 °C for 31 s.

### The specificity and sensitivity analysis of PEDV real-time RT-RPA method

A total of 10 ng of RNA or DNA extracted from PEDV DX, CV777, and JS2008, and TGEV, PKV, PDCoV, PPV, and PCV-2 was used as a template to analyze the specificity of PEDV real-time RT-RPA method on an Agilent Mx3000P thermocycler machine (Life Technologies). This was repeated three times.

Sensitivity analysis of two PEDV real-time RT-RPA assays was conducted using 10-serial dilutions of standard RNA as the original template ranging from 3 × 10^8^ to 3 × 10^1^ copies. A total of 1 μL of each serial dilution was used to evaluate the dynamic detection range of the two PEDV real-time RT-RPA assays. Meanwhile, 10^3.5^ TCID_50_/100 μL of classical attenuated vaccine strain CV777, 10^4.1^ TCID_50_/100 μL of Vero-cell-adapted isolate JS2008, and 10^5.2^ TCID_50_/100 μL wild DX strain were 10-fold serially diluted with Modified Eagle medium (MEM) and used as original templates in the RT-RPA method to determine the detection limitation. Each run was repeated four times, and a probabilistic regression analysis was performed with an Agilent Mx3000P thermocycler machine (Life Technologies) to determine the limits of the assay. The standard curve was calculated using GraphPad Prism 5.0 software (GraphPad Software Inc., San Diego, CA, USA).

### Clinical specimen test verification

Two pairs of primers, F1-V/R1 and F1-C/R1 [32], were used for RT-PCR with the PrimeScript™ One Step RT-PCR Kit Ver. 2.0 (Takara Co., Ltd., Dalian, China) to detect 80 samples with suspected PEDV infection from four locations in Gansu Province, China. The 80 fecal samples were obtained from five pig farms that have a history of inoculating the attenuated vaccine CV777, six of which from the piglets orally inoculated with the attenuated vaccine CV777 and show no clinical symptoms of diarrhea, and other 74 fecal samples were collected from piglets with symptoms of diarrhea. All fecal samples underwent PEDV real-time RT-RPA, PEDV real-time RT-PCR, and RT-PCR assays. Finally, all PEDV-positive products were sequenced by TsingKe Biological Technology.

## Supplementary information

**Additional file 1 **: **Table S1**. The name, accession number, and search website of the PEDV strain in the study.

## Data Availability

All data generated or analysed during this study are included in this published article (and its additional file table S1).

## Abbreviations

PEDV: Porcine epidemic diarrhea virus
PED: Porcine epidemic diarrhea
PCV-2: Porcine circovirus type 2
PDCoV: Porcine deltacoronavirus
PKV: Porcine kobuvirus
PPV: Porcine parvovirus
TGEV: Transmissible gastroenteritis virus
N: Nucleocapsid
S: Spike
ORF3: Open reading frame 3
E: Envelope
M: Membrane
nsp3: non-structural protein
S-INDEL: Insertion and deletion in the S gene
TCID_50_: 50% Tissue culture infective dose
CV: Coefficients of variation
SD: Standard deviation
RT-RPA: Reverse transcription recombinase polymerase amplification
RT-PCR: Reverse transcription-polymerase chain reaction
PCR: Polymerase chain reaction
