# Supplementary material for: Rapid differentiation of PEDV wild-type strains and classical attenuated vaccine strains by fluorescent probe-based reverse transcription recombinase polymerase amplification assay
Source: BMC Vet Res. 2020 Jun 22;16:208. doi: 10.1186/s12917-020-02424-1 (PMC7306936; doi:10.1186/s12917-020-02424-1)
Supplement: Supplementary file 1 — Additional file 1 : Table S1. The name, accession number, and search website of the PEDV strain in the study. [file 12917_2020_2424_MOESM1_ESM.pdf]

The name, accession number, and search website of the PEDV strain in the study

PEDV strain	Accession Number	search website
classical attenuated vaccine strain CV777	KT323979	https://www.ncbi.nlm.nih.gov/nuccore/KT323979
attenuated vaccine strain DR13	JQ023162	https://www.ncbi.nlm.nih.gov/nuccore/JQ023162
attenuated vaccine strain KC189944	KC189944	https://www.ncbi.nlm.nih.gov/nuccore/KC189944
JS2008	KC109141	https://www.ncbi.nlm.nih.gov/nuccore/KC109141.1
JS2008	KC210146	https://www.ncbi.nlm.nih.gov/nuccore/KC210146.1
SDM	JX560761	https://www.ncbi.nlm.nih.gov/nuccore/JX560761
HLJBY	KP403802	https://www.ncbi.nlm.nih.gov/nuccore/KP403802
SC1402	KP162057	https://www.ncbi.nlm.nih.gov/nuccore/KP162057
SQ2014	KP728470	https://www.ncbi.nlm.nih.gov/nuccore/KP728470
AVCT12	LC053455	https://www.ncbi.nlm.nih.gov/nuccore/LC053455
CH/M2013	KM887144	https://www.ncbi.nlm.nih.gov/nuccore/KM887144
SM98	GU937797	https://www.ncbi.nlm.nih.gov/nuccore/GU937797
LZC	EF185992	https://www.ncbi.nlm.nih.gov/nuccore/EF185992
EAS1	KR610991	https://www.ncbi.nlm.nih.gov/nuccore/KR610991
GDS01	KM089829	https://www.ncbi.nlm.nih.gov/nuccore/KM089829
CHGD-01	JX261936	https://www.ncbi.nlm.nih.gov/nuccore/JX261936
PEDV-7C	KM609204	https://www.ncbi.nlm.nih.gov/nuccore/KM609204
ZJCZ4	JX524137	https://www.ncbi.nlm.nih.gov/nuccore/JX524137
SH	MK841494	https://www.ncbi.nlm.nih.gov/nuccore/MK841494
PC21A	KR078299	https://www.ncbi.nlm.nih.gov/nuccore/KR078299
CV777	AF353511	https://www.ncbi.nlm.nih.gov/nuccore/AF353511
DR13	JQ023161	https://www.ncbi.nlm.nih.gov/nuccore/JQ023161
CH/HNAY/2015	KR809885	https://www.ncbi.nlm.nih.gov/nuccore/KR809885
XY2013	KR818832	https://www.ncbi.nlm.nih.gov/nuccore/KR818832
CHHNQX-314	KR095279	https://www.ncbi.nlm.nih.gov/nuccore/KR095279

CH/HNYF/14	KP890336	https://www.ncbi.nlm.nih.gov/nuccore/KP890336
PEDV-10F	KM609206	https://www.ncbi.nlm.nih.gov/nuccore/KM609206
PEDV-14	KM609207	https://www.ncbi.nlm.nih.gov/nuccore/KM609207
JSHA2013	KR818833	https://www.ncbi.nlm.nih.gov/nuccore/KR818833
JS-HZ2012	KC210147	https://www.ncbi.nlm.nih.gov/nuccore/KC210147
CHZJCX-12012	KF840537	https://www.ncbi.nlm.nih.gov/nuccore/KF840537
LZW isolate FGE_20140427	KJ777677	https://www.ncbi.nlm.nih.gov/nuccore/KJ777677
BJ-2011-1	JN825712	https://www.ncbi.nlm.nih.gov/nuccore/JN825712
PEDV-8C	KM609205	https://www.ncbi.nlm.nih.gov/nuccore/KM609205
AH2012	KC210145	https://www.ncbi.nlm.nih.gov/nuccore/KC210145
CH/JX-2/2013	KJ526096	https://www.ncbi.nlm.nih.gov/nuccore/KJ526096
PEDV-CHZ	KM609209	https://www.ncbi.nlm.nih.gov/nuccore/KM609209
LNCT2	KT323980	https://www.ncbi.nlm.nih.gov/nuccore/KT323980
CH/FJND-3/2011	JQ282909	https://www.ncbi.nlm.nih.gov/nuccore/JQ282909
CH/GDZQ/2014	KM242131	https://www.ncbi.nlm.nih.gov/nuccore/KM242131
PEDV-WS	KM609213	https://www.ncbi.nlm.nih.gov/nuccore/KM609213
CH/ZMDZY/11	KC196276	https://www.ncbi.nlm.nih.gov/nuccore/KC196276
CH/YNKM-8/2013	KF761675	https://www.ncbi.nlm.nih.gov/nuccore/KF761675
FL2013	KP765609	https://www.ncbi.nlm.nih.gov/nuccore/KP765609
LC	JX489155	https://www.ncbi.nlm.nih.gov/nuccore/JX489155
AJ1102	JX188454	https://www.ncbi.nlm.nih.gov/nuccore/JX188454
YN1	KT021227	https://www.ncbi.nlm.nih.gov/nuccore/KT021227

The name and accession number of the PEDV strains in figure 1 of the study. Everyone can find information of the PEDV strains by this website
